# Chemical Constituents from *Licania cruegeriana* and Their Cardiovascular and Antiplatelet Effects

**DOI:** 10.3390/molecules191221215

**Published:** 2014-12-17

**Authors:** Omar Estrada, Whendy Contreras, Giovana Acha, Eva Lucena, Whitney Venturini, Alfonso Cardozo, Claudia Alvarado-Castillo

**Affiliations:** 1 Centro de Biofisica y Bioquímica, Instituto Venezolano de Investigaciones Científicas (IVIC), Altos de Pipe 1020-A, Venezuela; E-Mails: oestrada@ivic.gob.ve (O.E.); wcontreras@ivic.gob.ve (W.C.); gacha@ivic.gob.ve (G.A.); elucena@ivic.gob.ve (E.L.); wventurini@ivic.gob.ve (W.V.); 2 Facultad de Agronomía, Universidad Central de Venezuela, Maracay 2101, Venezuela; E-Mail: alfonosocardozo@gmail.com

**Keywords:** lupane-type triterpenoids, *Licania cruegeriana*, * platelet aggregation*, SHR

## Abstract

Three new lupane-type triterpenoids: 6β,30-dihydroxybetulinic acid glucopyranosyl ester (**4**), 6β,30-dihydroxybetulinic acid (**5**) and 6β-hydroxybetulinic acid (**6**), were isolated from *Licania cruegeriana* Urb. along with six known compounds. Their structures were elucidated on the basis of spectroscopic methods, including IR, ESIMS, 1D- and 2D-NMR experiments, as well as by comparison of their spectral data with those of related compounds. All compounds were evaluated* in vivo* for their effects on the mean arterial blood pressure (MABP) and heart rate (HR) of spontaneously hypertensive rats (SHR) and also* in vitro* for their capacity to inhibit the human platelet aggregation. None of the isolated flavonoids **1**–**3** showed cardiovascular effects on SHR and among the isolated triterpenoids **4**–**9** only **5** and **6** produced a significant reduction in MABP (60.1% and 17.2%, respectively) and an elevation in HR (11.0% and 41.2%, respectively). Compounds **3**, **4**, **5** and **6** were able to inhibit human platelet aggregation induced by ADP, collagen and arachidonic acid with different selectivity profiles.

## 1. Introduction

The genus Licania (Chrysobalanaceae) consists of more than 200 species of trees and shrubs, which are mainly distributed in tropical regions of America and Africa [1,2]. The species of the genus *Licania* have been used in South America for various medicinal purposes such as the treatment of inflammation [3], diabetes [4], stomach ache, diarrhea, and dysentery [5]. Previous phytochemical studies of this genus have reported the isolation of two main classes of compounds: flavonoid glycosides based on myricetin and quercetin moieties and triterpenes of the lupane, oleane or ursane types [4]. In the present work, we report the isolation from the leaves of *Licania cruegeriana* Urb. and structure elucidation of three new lupane-type triterpenoids: 6β,30-dihydroxybetulinic acid gluco-pyranosyl ester (**4**) 6β,30-dihydroxybetulinic acid (**5**) and 6β-hydroxybetulinic acid (**6**), along with six known compounds. Additionally, all isolated compounds were evaluated for their effects on the mean arterial blood pressure (MABP) and heart rate (HR) of spontaneously hypertensive rats (SHR) and also for their capacity to inhibit the human platelet aggregation *in vitro*.

## 2. Results and Discussion

### 2.1. Extraction and Isolation

**Figure 1:**
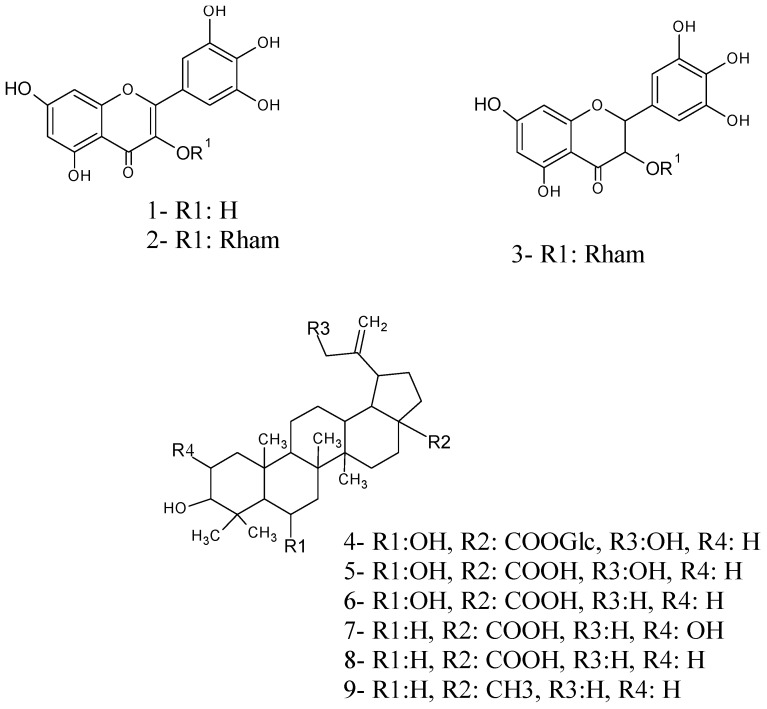
Structures of isolated compounds from *Licania cruegeriana*.

Fresh leaves (310 g) were extracted by percolation with ethanol for a week. The solvent was evaporated *in vacuo* to yield 89.0 g of ethanolic extract (EE). Two fractions were obtained from EE partition in methanol-water (1:1): A red solution that was then evaporated *in vacuo* to yield a red residue (16.7 g), named methanol-water soluble fraction (MWSF), and a green residue (71.2 g) named methanol-water insoluble fraction (MWIF). A portion of MWSF (5.0 g) was three times extracted with acetone to obtain a brownish residue and a yellowish solution that was then concentrated to dryness yielding a yellowish residue named AF (4.7 g). AF (1 g) was fractionated on Sephadex LH-20 column chromatography (CC) using methanol as eluent to give three fractions named I–III. Myricetin (**1**, 200 mg) was separated from fraction III by CC on RP-18, eluting with a mixture methanol-water (3:2). From fraction II myricetin 3-*O*-α-rhamnoside (**2**, 100 mg) and dihydromyricetin-3-*O*-α-rhamnoside (**3**, 180 mg) were separated by low-pressure CC with a mixture of acetonitrile-water (2:3). MWIF (2 g) was subjected to low-pressure CC with a mixture of methanol-water (7:3) as eluent to afford 6β,30-dihydroxybetulinic acid glucopyranosyl ester (**4**, 50 mg), 6β,30-dihydroxybetulinic acid (**5**, 100 mg), 6β-hydroxybetulinic acid (**6**, 320 mg), alphitolic acid (**7**, 20 mg), betulinic acid (**8**, 19 mg) and lupeol (**9**, 15 mg). The structure of compounds **1**–**3** and **7**–**9** (Figure 1) were established by comparing their ^1^H- and ^13^C-NMR chemical shifts and proton coupling constants with those previously reported in the literature [6,7,8,9], whereas the structure elucidation of compounds **4**, **5** and **6** is described below.

### 2.2. Structure Elucidation of Compounds **4**, **5** and **6**

The molecular formulae of compounds **5** and **6** were assigned as C_30_H_48_O_5_ and C_30_H_48_O_4_, respectively, from their ESIMS and NMR data. The analysis of their ^1^H- and ^13^C-NMR spectra indicated that these compounds are lupane-type triterpenoids.

The Δ^20,29^-functionality of the lupene skeletons were inferred from the resonances of the sp^2^ carbons at C-29 (secondary carbon signal deduced by DEPT pulse sequence) at δ*_C_* 106.9 and δ*_C_* 110.2 ppm and C-20 (quarternary carbon) at δ*_C_* 156.3 and δ*_C_* 152.0 ppm of **5** and **6** respectively [8]. From the ^13^C-NMR spectra is was deduced that these triterpenoids have a hydroxyl group at C-6 by the shifts of the ^13^C-NMR signals at δ*_C_* 19.7 ppm (C-6) of betulinic acid [8] to δ*_C_* 69.3 ppm in **5** and **6**. In both compounds the axial orientation of the hydroxyl group at C-6 was inferred by the shifts of the ^13^C-NMR signal at δ*_C_* 16.4 ppm (C-24) in 6α-hydroxybetulinic acid [9] to δ*_C_* 24.8 ppm, thus compound **6** was deduced as 6β-hydroxybetulinic acid. 

**Figure 2:**
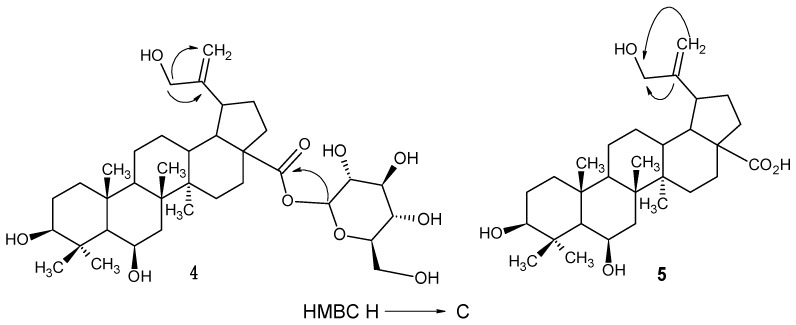
Key HMBC correlations observed for compounds **4** and **5**.

The signals in the ^13^C-NMR spectra of **5** and **6** are similar, except those at C-30 and those corresponding to the proton coupling patterns. The shifts of the signals at δ*_C_* 19.6 (C-30) in **6** to δ*_C_* 65.2 in **5** suggest an additional hydroxyl substituent at this position which was confirmed in the HMBC spectrum of **5** given the correlations between H-29a/H29b (δ_H_ 4.96, 4.85) and the carbon signal at δ*_C_* 65.2 ppm and also between H-30 at δ_H_ 4.0 ppm and C-20 at δ*_C_* 156.3 ppm (Figure 2), indicating that a hydroxyl group is located at C-30 in **5**. Thus, the structure of **5** was determined to be 6β,30-dihydroxybetulinic acid.

Compound **4** has a molecular formula of C_36_H_58_O_10_ according to ESIMS (*m/z* 649.4 [M−H]^−^). The comparison of ^1^H- and ^13^C-NMR spectroscopic data of **4** with those of **5** indicated that **4** is the glycosylated derivative of **5**. The NMR spectra of **4** showed an anomeric proton at δ_H_ 5.48 ppm (d, *J_H_*_1',H2'_ = 8.0 Hz) with the corresponding carbon at δ*_C_* 95.2 ppm. Comparison of NMR data with those reported in the literature suggested a β*-*oriented glucopyranoside moiety on the basis of the large ^3^*J*_H1',H2'_ coupling constant [10,11,12,13,14,15,16,17]. The HMBC correlations of the anomeric proton H-1' to C-28 (δ*_C_* 176.2) indicated that the β-glucopyranosyl unit was attached to the carboxyl group. Thus, the structure of **4** was determined to be 6β,30-dihydroxybetulinic acid glucopyranosyl ester. To the best of our knowledge this is the first report of compounds **4**, **5** and **6** from Nature.

### 2.3. Cardiovascular Effects of the Isolated Compounds

To determine the cardiovascular effects of the isolated compounds from *L. cruegeriana*, all of them were intravenously administered to pentobarbital-anaesthetized SHR over thirty seconds and the MABP and the HR were monitored continuously during forty-five minutes as described in Experimental Section 3.4.1. None of the isolated flavonoids showed cardiovascular effects in SHR and among the isolated triterpenoids only compounds **5** and **6** induced changes in MABP and HR of SHR as shown in Table 1. Compounds **5** and **6** produced significant reductions in MABP (60.1% and 17.2%, respectively) and induced elevation in HR (11.0% and 41.2%, respectively). These cardiovascular effects exhibit a time of peak effect of three minutes and were recorded for more than forty-five minutes without recovering baseline levels. The widely used antihypertensive drug losartan (an AT1 receptor antagonist) served as a positive control [18,19,20]. In the present study, losartan injection (0.3 mg/kg) reduced MABP by 19.3% and increased HR by 27.7% (*p* < 0.05). The reduction in MABP lasted longer than forty-five minutes and showed a time of peak effect near ten minutes.

Analysis of the relationships between the molecular structures of the cardiovascular active compounds **5** and **6** and their structurally related compounds **4**, **7**, **8** and **9** (Figure 1), led to conclude that the substitution of carboxylic group in **4** with a glycoside seems to interfere with the cardiovascular active motive of this compound and that the hydroxylation of the betulinic moieties at C-6 and C-30 enhance its cardiovascular effects.

### 2.4. Antiplatelet Effects of Isolated Compounds

In Table 2 are shown the isolated compounds from *L. cruegeriana* which were able to inhibit the aggregation of human platelets induced by arachidonic acid (AA), collagen and adenosine 5'-diphosphate (ADP). 

**Table 1:** Effects of compounds **5** and **6** isolated from *Licania cruegeriana* on the mean arterial blood pressure (MABP) and heart rate (HR) of spontaneously hypertensive rats (SHR).

Compounds	MABP (mmHg) Basal	MABP (mmHg) After Treatment	Maximal (%) Change	HR (bpm) Basal	HR (bpm) after Treatment	Maximal (%) Change	Time Peak (s)	Time Recovery (min)
**Vehicle**	141 ± 8	137 ± 5	−3.1	422 ± 21	414 ± 12	−1.8	-	-
**5**	138 ± 5	55 ± 10 **	−60.1 **	290 ± 15	330 ± 12 *	11.0	180 ± 20	>45
**6**	145 ± 10	120 ± 12 *	−17.2 *	405 ± 18	572 ± 16 **	41.2	180 ± 10	>45
**Losartan**	145 ± 12	117 ± 9 *	−19.3 *	350 ± 32	447 ± 21 **	27.7	600 ± 9	>45

Compounds **5**, **6** and losartan at 0.3 mg/kg were i.v. injected in 0.1 mL of 5% DMSO in physiological saline solution (vehicle) through the femoral vein of anesthetized SHR over 30 seconds. Increases (+) and decreases (−) in MABP and HR are indicated in the maximal percent of change columns. For each MABP variation, the time of peak effect and complete recovery to basal values are given. The time of peak effect was measured from the beginning of the injection. Values are the mean ± S.D (*n* = 4 of each). * *p* < 0.05 and ** *p* < 0.01 *vs.* basal values when unpaired Student’s test was applied. One-way ANOVA test, comparing basal values between treatment groups of MABP and HR showed no significant differences among them (*p* > 0.05 for both).

**Table 2:** Effects of compounds **3**, **4**, **5** and **6** on human platelet aggregation.

Compounds	Aggregation (%)		
	AA	Collagen	ADP
Control	100	100	100
**3**	1.2 ± 0 ***	18.7 ± 6 **	40.1 ± 6 *
**4**	49.5 ± 6	13.6 ± 6 ***	90.6 ± 5
**5**	87.5 ± 2	18.5 ± 7 **	22.9 ± 5 **
**6**	89.1 ± 4	32.5 ± 6 *	61.6 ± 5

Platelets in PRP were preincubated with each compound at 250 µg/mL, which correspond to 536 µM (**3)**, 385 µM (**4)**, 510 µM (**5)** and 530 µM (**6)**, or 0.25% DMSO (control) for 15 min, then platelet aggregation was stimulated by addition of AA (0.5 mM), collagen (1.5 µg/mL) or ADP (5 μM), at 37 °C under 1000 rpm stirring. Values are presented as mean ± S.E. of (*n 5–*6) of the percent of aggregation response compared to their respective controls. One way ANOVA Kruskal-Wallis test and Dunn’s multiple comparisons test (* *p* < 0.05, ** *p* < 0.01, and *** *p* < 0.001 *vs.* control) were applied.

Compound **3** was the only isolated flavonoid that exhibited antiplatelet effects causing total inhibition against the action of AA, a significant decrease against collagen and near middle inhibition against ADP. Among the isolated triterpenoids, compound **4** significantly inhibited the aggregation of platelets induced by collagen and had an average effect against the actions of AA, and compound **5** significantly decreased the ADP and collagen-induced platelet aggregations, while compound **6** exhibited significant antiplatelet effect against collagen. The mode of action by which these compounds exert their effects on platelet aggregation needs to be further studied.

## 3. Experimental Section

### 3.1. General Information

RediSep^®^ Rf Reversed-phase C18 was used for low pressure CC and silica gel 60 RP18 F254 (E. Merck) for TLC on glass (Merck). Dimethylsulfoxide (DMSO), adenosine 5'-diphosphate sodium salt (ADP) and Sephadex^®^LH-20 were obtained from Sigma Aldrich (St. Louis, MO, USA). ^1^H- and ^13^C-NMR spectra were obtained using a Bruker DRX 500 (500 MHz for ^1^H and 125 MHz for ^13^C) and Bruker Avance 300 (300 MHz for ^1^H and 75 MHz for ^13^C) in CD_3_OD. Measurements of electrospray ionization mass spectra were acquired in negative ion mode on an Ion Trap mass spectrometer (Amazon SL, Bruker, Bremen, Germany). Infrared (IR) spectra (KBr discs) were recorded using a FTIR spectrophotometer (Perkin Elmer, Shelton, CT, USA). Collagen and arachidonic acid were from Helena Laboratories (Beaumont, TX, USA). All solvents used were of HPLC grade quality, obtained commercially from Sigma. The purity of compounds **4**, **5** and **6** was confirmed by using an HPLC-MS Agilent 1260 series LC/MSD trap, SL model (Bruker) equipped with an electrospray interface (ESI), a quaternary pump, degasser, autosampler and a thermostatted column compartment. A column XBridge^TM^ C18 4.6 × 75 mm, 2.5 μm (Waters, Dublin, Ireland) was used and kept at 25 °C in the column compartment. Nitrogen was used as nebulizing and drying gas at 220 °C. The ESI source was operated in negative ion mode. Complete system control, data acquisition and processing were done using the HyStar 3.2 for LC/MSD trap software from Bruker. The injection volume was 5 μL. The mobile phase was delivered in isocratic mode and consisted of a mixture of methanol/water (70:30). The chromatograms were recorded in full scan mode for compounds **4**, **5** and **6** and only MS/MS mode for compound **4** (in negative mode MW = 649). Full-scan spectra were acquired over a scan range of *m/z* 70–2200 at 32.5 *m/z/*s.

### 3.2. Plant Material

The leaves of *Licania cruegeriana Urb.* were collected in August 2009 at the Parque Nacional Henri Pittier, Aragua, Venezuela. This specimen was identified by Dr. Alfonso Cardozo and a voucher specimen (AC2706) was deposited in the herbarium of Facultad de Agronomía, UCV, Maracay.

### 3.3. Spectral Data

*3β,6β,30-Trihydroxy-20(29)-lupen-28-O-β-glucopyranosyl ester* (**4**). A white powder, IR (KBr) υ_max_ 2947, 2871, 1705, 1651, 1386, 1187, 1059 cm^−1^; ^1^H-NMR (CD_3_OD, 500 MHz): δ 5.48 (1H, *d*, *J =* 8 Hz, H-1'), 4.96 (1H, *br s*, H-29a), 4.85 (H-29b, under CD_3_OD signal), 4.32 (1H, *br s*, H-6), 4.04 (2H, *br s*, H-30), 3.82 (1H, *d*, *J* = 12Hz, H-6'a), 3.70 (1H, *d*, *J* = 12Hz, H-6'b), 3.45 (*m*, H-3'), 3.37 (*m*, H-4'), 3.31(*m*, H-2'), 3.25 (1H, *br s*, H-3) , 2.88 (1H, *m*, H-19), 2.37 (2H, *m*, H-13 and H-16), 2.03 (3H, *m*, H-12 and H-21), 1.8 (1H, *t*, *J* = 11.5 Hz, H-9), 1.6 (*m*, H-7), 1.25 (3H, *s*, Me-26), 1.20 (3H, *s*, Me-24), 1.18 (3H, *s*, Me-25), 0.99 (3H, *s*, Me-27), 0.97 (3H, *s*, Me-23); ^13^C-NMR (CD_3_OD, 125 MHz,): δ 176.2 (C-28), 156.2 (C-20), 107.1 (C-29), 95.2 (C-1'), 78.7 (C-5'), 78.5 (C-3), 78.3 (C-3'), 74.0 (C-2'), 71.1 (C-4'), 69.3 (C-6), 65.3 (C-30), 62.3 (C-6'), 58.0 (C-17), 52.2 (C-5), 51.2 (C-9), 50.1 (C-18), 43.9 (C-19), 43.7 (C-14), 42.8 (C-7), 41.3 (C-8), 39.4 (C-4), 38.4 (C-13), 38.0 (C-10), 37.3 (C-21), 36.8 (C-21), 33.2 (C-16), 32.6 (C-22), 30.9 (C-15), 28.9 (C-23), 28.1 (C-2), 26.3 (C-12), 24.8 (C-24), 22.1 (C-11), 18.0 (C-25), 17.3 (C-26), 15.4 (C-27). Negative mode ESI-MS: *m/z* 649.4.

*3β,6β,30-Trihydroxy-20(29)-lupen-28-oic-acid* (**5**). A white powder, IR (KBr) υ_max_ 2946, 2870, 1704, 1645, 1452, 1397, 1202, 1058 cm^−1^; ^1^H-NMR (CD_3_OD, 300 MHz): δ 4.96 (1H, *br s*, H-29a), 4.33 (1H, *m*, H-6), 4.04 (2H, *br s*, H_2_-30), 3.25 (1H, *m*, H-3), 2.90 (1H, *m*, H-19), 1.26 (3H, *s*, Me-26), 1.20 (3H, *s*, Me-25), 1.19 (3H, *s*, Me-24), 1.0 (3H, *s*, Me-23), 0.97 (3H, *s*, Me-27); ^13^C-NMR (CD_3_OD, 75 MHz): δ 180.0 (C-28), 156.3 (C-20), 106.9 (C-29), 78.5 (C-3), 69.3 (C-6), 65.2 (C-30), 57.5 (C-17), 52.1 (C-5), 51.1 (C-9), 50.2 (C-18), 44.1 (C-19), 43.7 (C-14), 42.8 (C-7), 41.2 (C-8), 39.4 (C-4), 38.7 (C-13), 38.0 (C-10), 37.9 (C-1), 36.8 (C-21), 33.4 (C-16), 33.1 (C-22), 31.0 (C-15), 28.9 (C-23), 28.1 (C-2), 26.3 (C-12), 24.8 (C-24), 22.1 (C-11), 18.0 (C-25), 17.3 (C-26), 15.4 (C-27). Negative mode ESI-MS: *m/z* 487.5.

*3β,6β-Dihydroxy-20(29)-lupen-28-oic-acid* (**6**). A white powder, IR (KBr) υ_max_ 2920, 2870, 1700, 1642, 1452, 1394, 1180, 1064 cm^−1^; ^1^H-NMR (CD_3_OD, 300 MHz): δ 4.71 and 4.59 (2H, each *br s*, H-29a, and H-29b), 4.33 (1H, *br s*, H-6), 3.25 (1H, *m*, H-3), 3.05 (1H, *m*, H-19), 1.69 (6H, *br s*, Me-30 and Me-26), 1.26 (3H, *s*, Me-24), 1.20 (3H, s, Me-25), 1.19 (3H, s, Me-27), 0.97 (3H, s Me-23); ^13^C-NMR (CD_3_OD, 75 MHz): δ 180.1 (C-28), 152.0 (C-20), 110.2 (C-29), 78.5 (C-3), 69.3 (C-6), 57.5 (C-17), 52.1 (C-5), 50.5 (C-9), 50.2 (C-19), 48.4 (C-18), 43.8 (C-14), 42.8 (C-7), 41.2 (C-8), 39.3 (C-4) 38.8 (C-13), 38.1 (C-10), 38.0 (C-1), 36.8 (C-22), 33.1 (C-15), 31.7 (C-21), 30.9 (C-16), 28.9 (C-23), 27.0 (C-2), 26.3 (C-12), 24.8 (C-24), 22.0 (C-11), 19.6 (C-30), 18.1 (C-26), 17.3 (C-25), 15.4 (C-27). Negative mode ESI-MS *m*/*z:* 471.5.

### 3.4. Biological Assays

#### 3.4.1. Cardiovascular Assay

Spontaneously Hypertensive Rats (SHR), male (250–300 g) were used for all experiments and were obtained from the animal care service of IVIC. Animals were housed under conditions of controlled temperature (21 ± 2 °C) and lighting (lights on 06:00–18:00 h). In addition, they had free access to food (RATARINA, Protinal, Maracay, Venezuela) and tap water. All animal procedures were approved by the bioethical committee of IVIC (number 201417). SHR were anesthetized by an intraperitoneal (i.p.) injection of sodium pentobarbital (40 mg/kg). The trachea was exposed and cannulated with a polyethylene catheter to avoid ventilation disturbances. Arterial blood pressure was recorded from the femoral artery through a catheter connected to a blood pressure transducer (MLT844, PowerLab, Melbourne, Australia) and a bridge amplifier (ML110, PowerLab) from which MABP and HR were continuously recorded using a 4/20 High Performance Data Recording System (PowerLab). To facilitate the intravenous (i.v.) administration of isolated compound from *L. cruegeriana,* an i.v. line was placed in the femoral vein using a polyethylene catheter. Once the basal conditions remained constant for more than 45 min, the changes in MABP and HR induced by the *L. cruegeriana* samples were recorded for at least 45 min after injection. Samples, at the indicated doses, were injected as a single bolus of 0.1 mL (5% DMSO in physiological saline solution, as vehicle) over 30 s. The doses of isolated compounds from *L. cruegeriana* used in this study were the minimal doses that after inducing a significant hypotensive effect allowed the survival of rats for at least 2 h. This protocol was evaluated and approved by the Bioethics Commission for Investigations in Animals (COBIANIM) at the Venezuelan Institute for Scientific Research (IVIC) (Protocol 201417, approval on November 2014), in accordance with the Code on Bioethics and Biosecurity (2008) established by the Bioethics Commission National Fund on Science and Technology (FONACIT), under the national legislation (LOCTI, 2005).

#### 3.4.2. *In Vitro* Platelet Aggregation Assay

Human platelets were obtained from blood of healthy volunteers who did not take any drugs during previous two weeks and gave informed consent before taking part in this study. The written informed consent form and this protocol were evaluated and approved by the Bioethics Commission for Investigations involving Human Subjects of the Venezuelan Institute for Scientific Research (IVIC) (Project identification code 1316, approval on March 2009), in accordance with the Code on Bioethics and Biosecurity (2008) established by the Bioethics Commission National Fund on Science and Technology (FONACIT), under the national legislation (LOCTI, 2005). Platelet rich plasma (PRP) was prepared and used in platelet aggregation assays as described earlier [21]. Inhibition experiments were done by incubating the platelets with 250 µg/mL of each isolated compound (for 15 min) before their stimulation by the addition of ADP (5 µM), collagen (1.5 µg/mL) and arachidonic acid (0.5 mM).

## 4. Conclusions

The present chemical investigation of the leaves of *L. cruegeriana* led to the isolation and identification of myricetin, two myricetin glycosides and six triterpenoids with lupane moieties (Figure 1). This constitutes the first phytochemical study for this species. To the best of our knowledge the following betulinic acid derivates: 6β,30-dihydroxybetulinic acid glucopyranosyl ester (**4**), 6β,30-dihydroxybetulinic acid (**5**) and 6β-hydroxybetulinic acid (**6**) have never been reported before in the literature. The myricetin derivates found in *L. cruegeriana* seem to follow a similar glycosylation pattern to those reported for the *Licania* genus and other species of the Chrysobalanaceae family [6], since two of them are glycosylated at C-3 having rhamnose as the common sugar. Of the six triterpenes **4**–**9** with lupane skeletons identified in *L. cruegeriana* only betulinic acid, lupeol and betulin have previously been reported in species of *Licania* [22]. 6β-Hydroxybetulinic acid was the major chemical constituent found in *L. cruegeriana* leaves.

Pharmacological studies of some plants growing in Venezuela are being conducted by our research group in order to identify secondary metabolites as potential therapeutic agents for cardiovascular diseases. In the present study, none of the flavonoids isolated from *L. cruegeriana* had cardiovascular effects in SHR and only two triterpenoids **5** and **6** showed significant reduction in MABP (60.1% and 17.2% respectively) inducing an elevation in HR (11.0% and 41.2% respectively) as shown in Table 1. A simple structure–activity relationship analysis of these data would suggest that when triterpenoids increase their oxidation state by introducing a hydroxyl group, it seems to be sufficient to significantly increase their hypotensive properties, which is in concordance with what is reported for triterpenoids such as pomolic acid [23] and ursolic acid [24]. On the other hand, one flavonoid (compound **3**) and three triterpenoids (compounds **4**, **5** and **6**) were able to inhibit the aggregation of human platelets induced by AA, collagen and ADP as shown in Table 2. These data would suggest that the absence of the double bound at C-2 in myricetin moiety is necessary for the antiplatelet effect of this kind of flavonoids. In the case of lupane-type triterpenoids **4**, **5** and **6** the addition of one hydroxyl group at C-30 in compound **4** and **5** appears to increase their antiplatelet properties against collagen stimulation. Additionally, the glycosylation at C-28 in compound **4** seems to decrease the anti-platelet effect against ADP with respect to compound **5**. None of these triterpenoids have significant effect on the AA-induced platelet aggregation.

Taken together, the present phytochemical study of *L. cruegeriana* leaves is a novel contribution to the current acknowledge of the phytochemistry of *Licania* genus, being remarkable that triterpenoids with a lupane-type skeletons and flavonoids, particularly myricetin and their glycosides are the characteristic chemotaxonomic markers for this genus [6]. Additionally, herein we report some new triterpenoids with pharmacological activities that might be useful for the treatment of cardiovascular diseases.

## Supplementary Materials

Supplementary materials can be accessed at: http://www.mdpi.com/1420-3049/19/12/21215/s1.
