# Supplementary material for: Chemical Constituents from Licania cruegeriana and Their Cardiovascular and Antiplatelet Effects
Source: Molecules. 2014 Dec 17;19(12):21215–25. doi: 10.3390/molecules191221215 (PMC6270790; doi:10.3390/molecules191221215)

Supplementary Materials

Figure S1. ^{1}H-NMR spectrum of compound **4**.

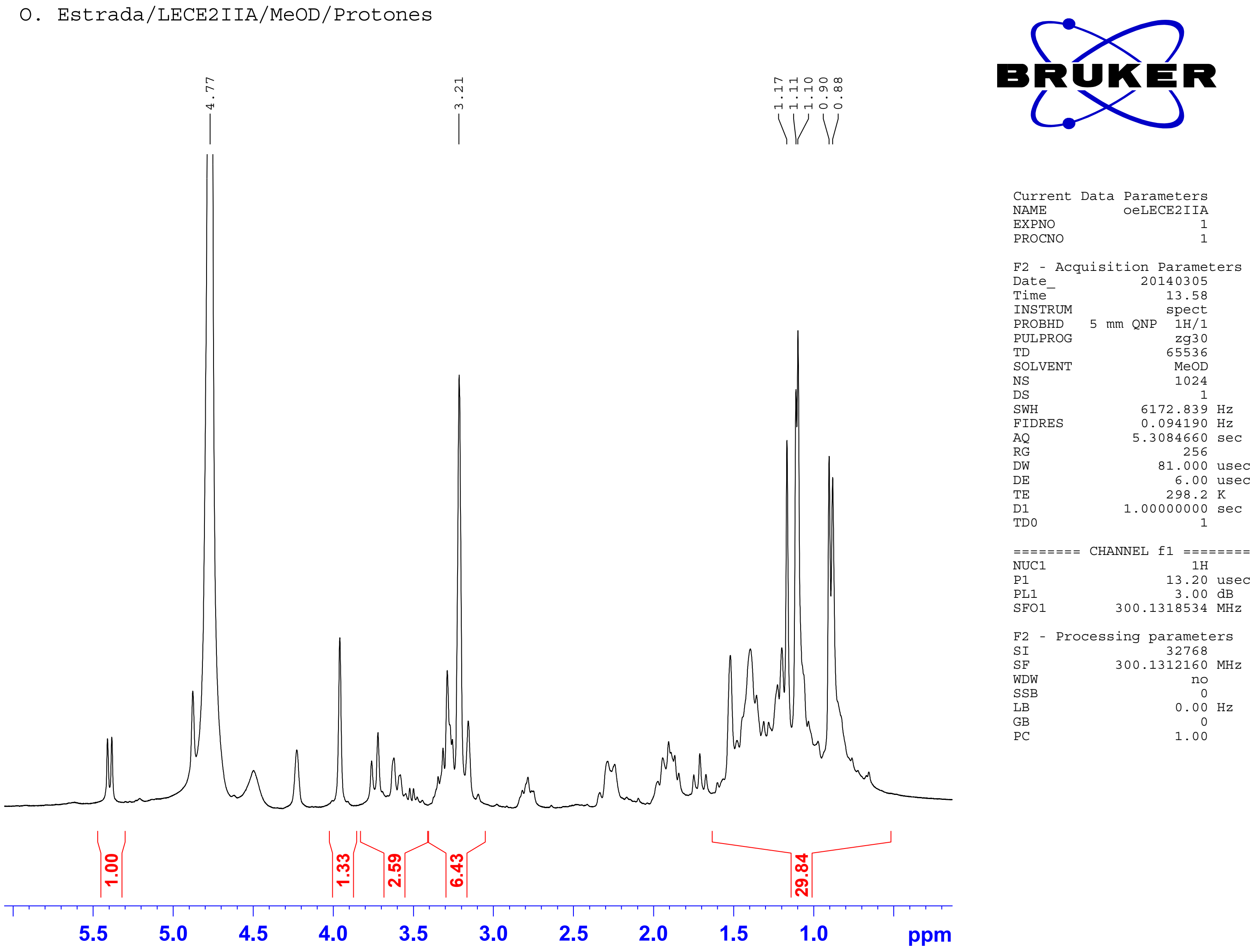

Figure S2. ^{13}C-NMR spectrum of compound **4**.

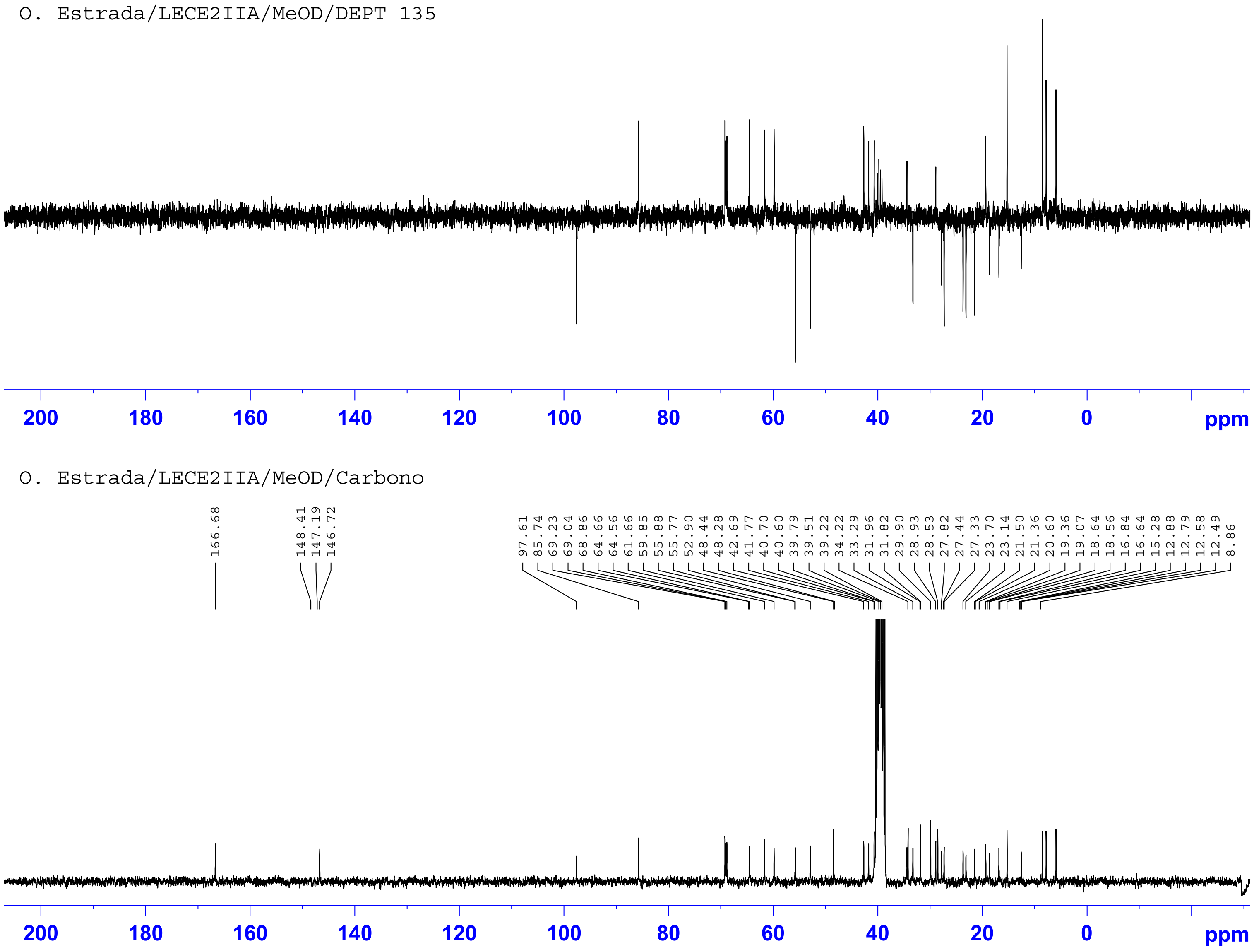

Figure S3. HMQC spectrum of compound **4**.

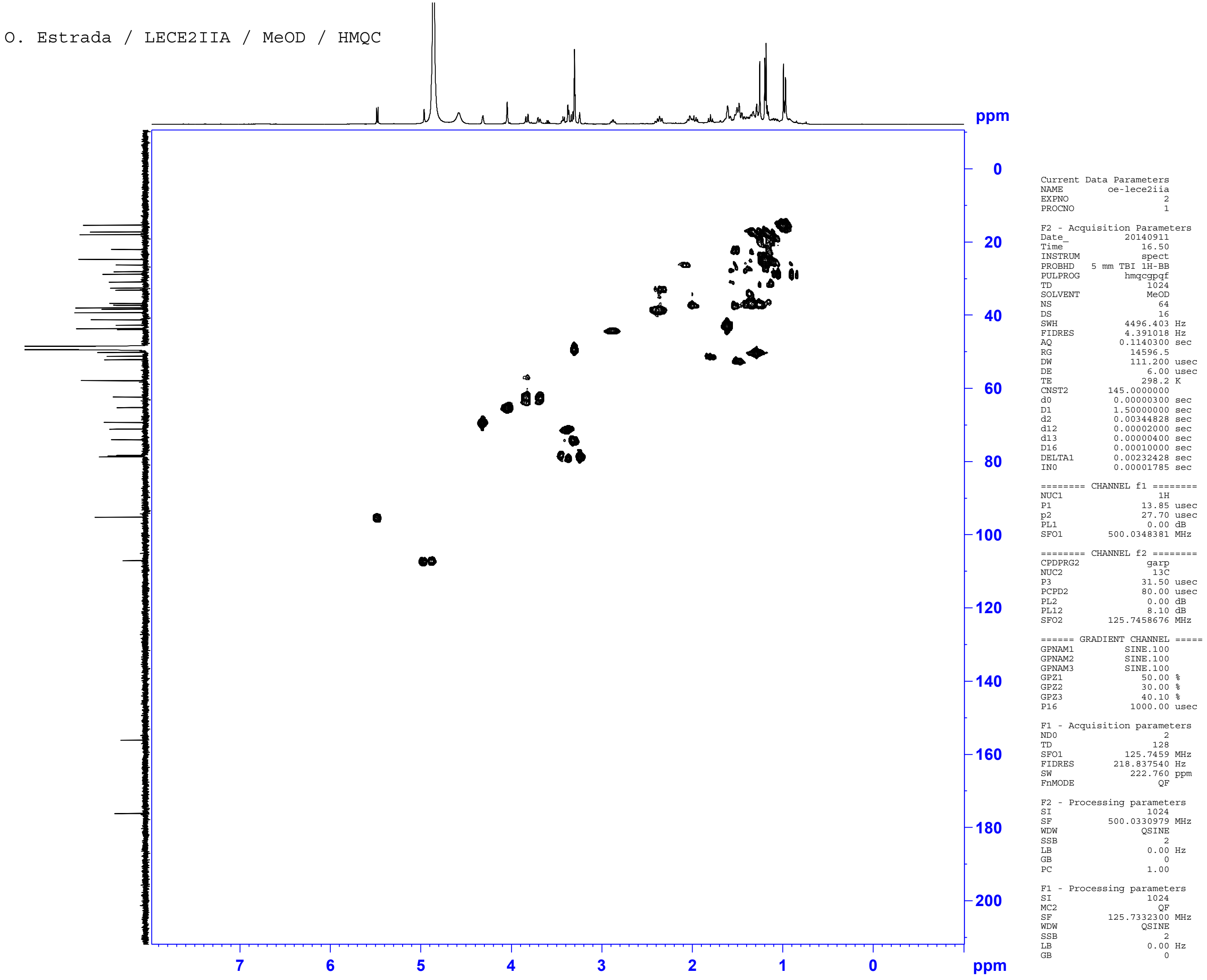

Figure S4. HMBC spectrum of compound **4**.

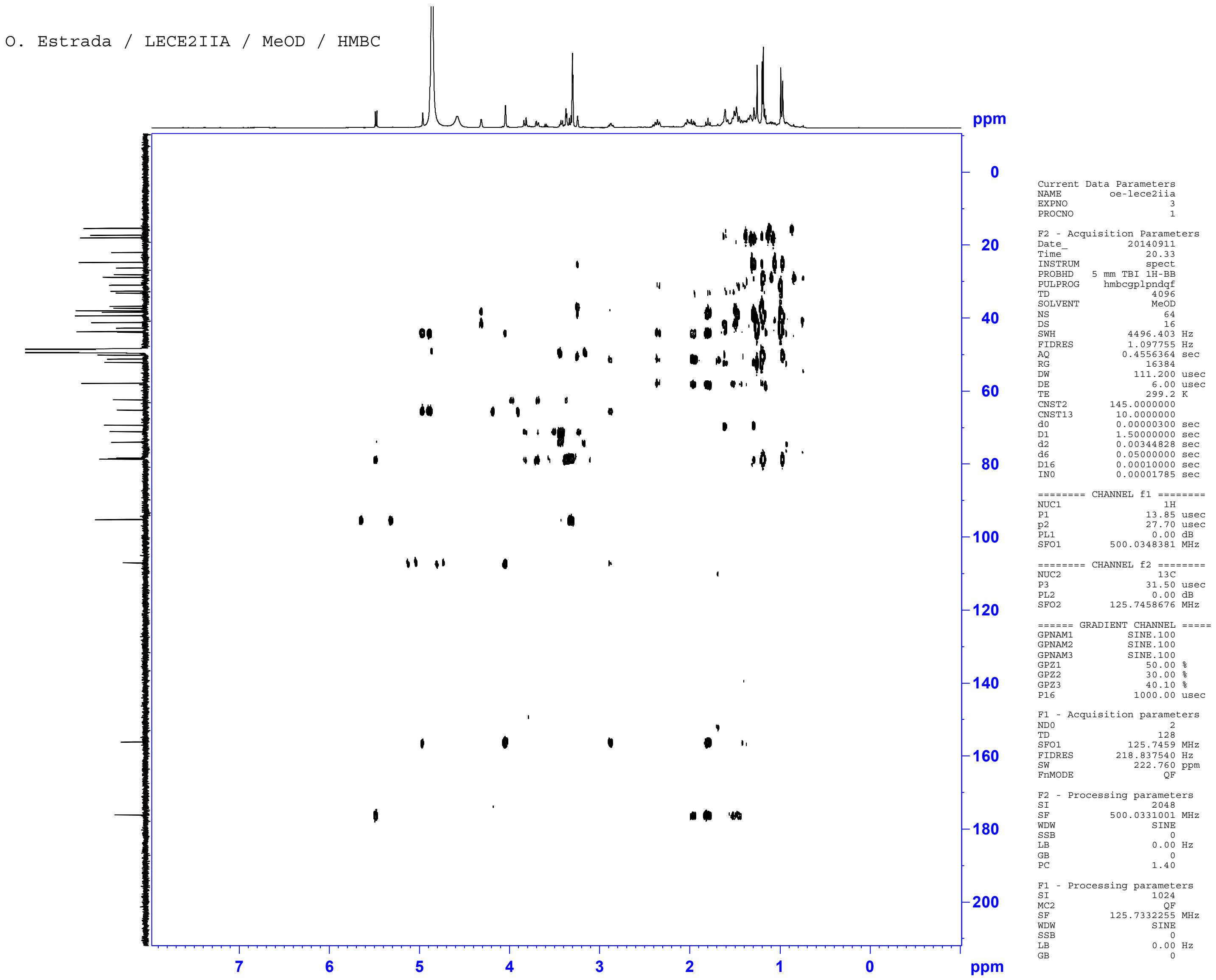

Figure S5. HPLC-MS-full scan of compound **4**.

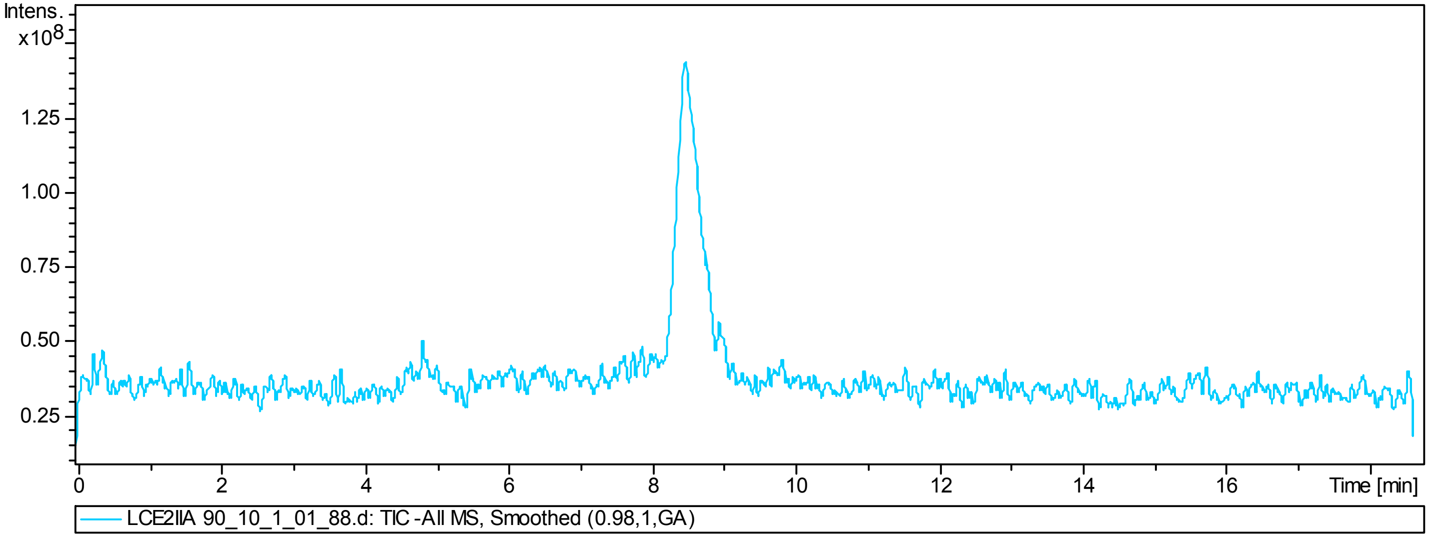

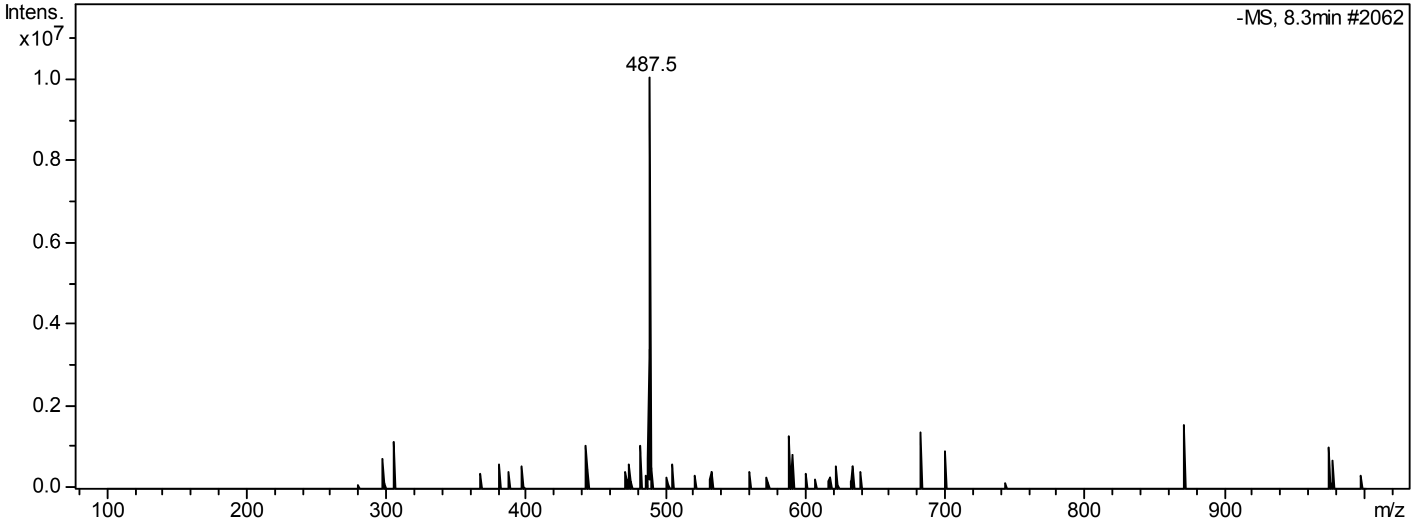

Figure S6. MS/MS 649 *m/z* of compound **4**.

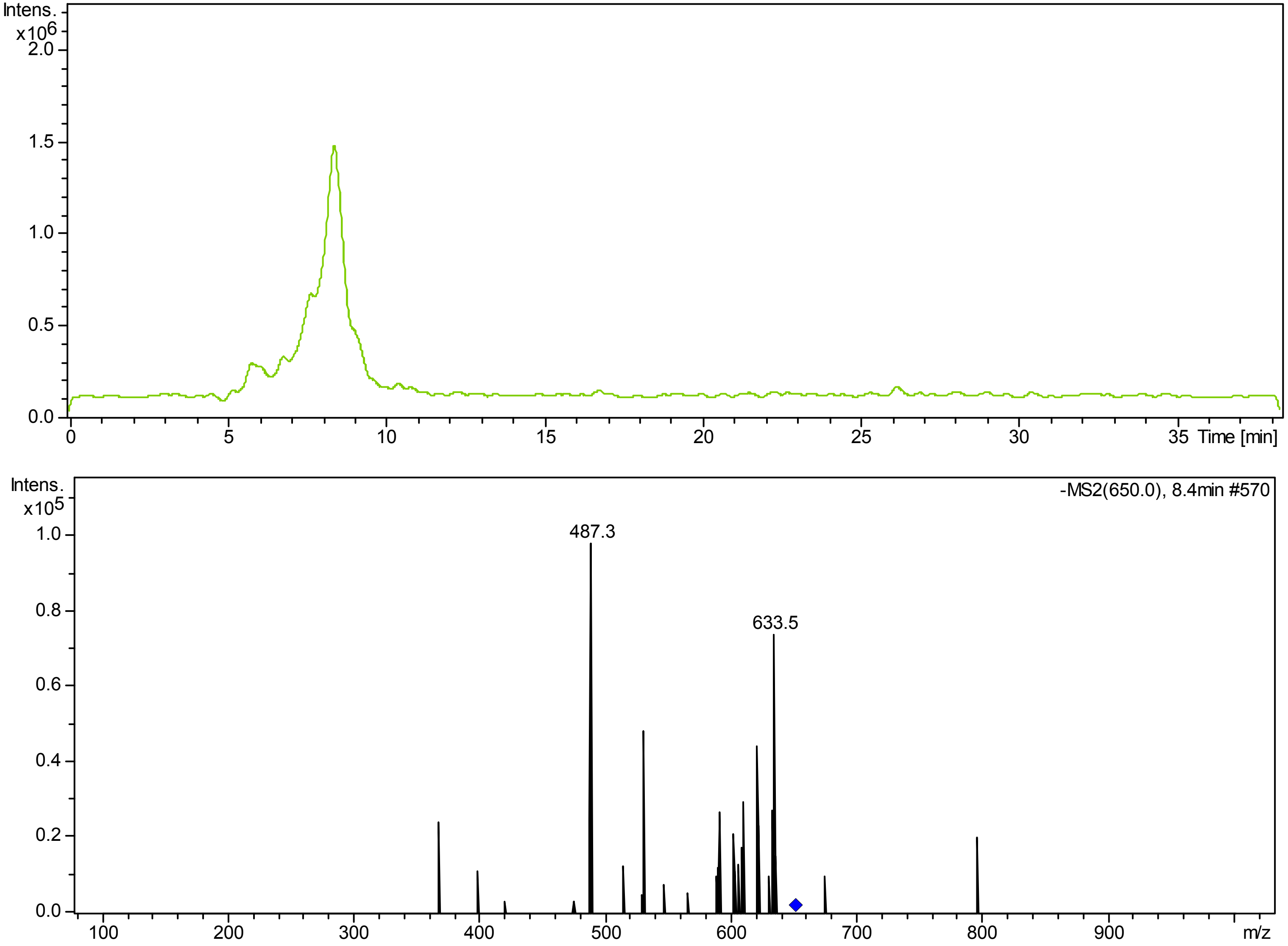

Figure S7. IR spectrum of compound **4**.

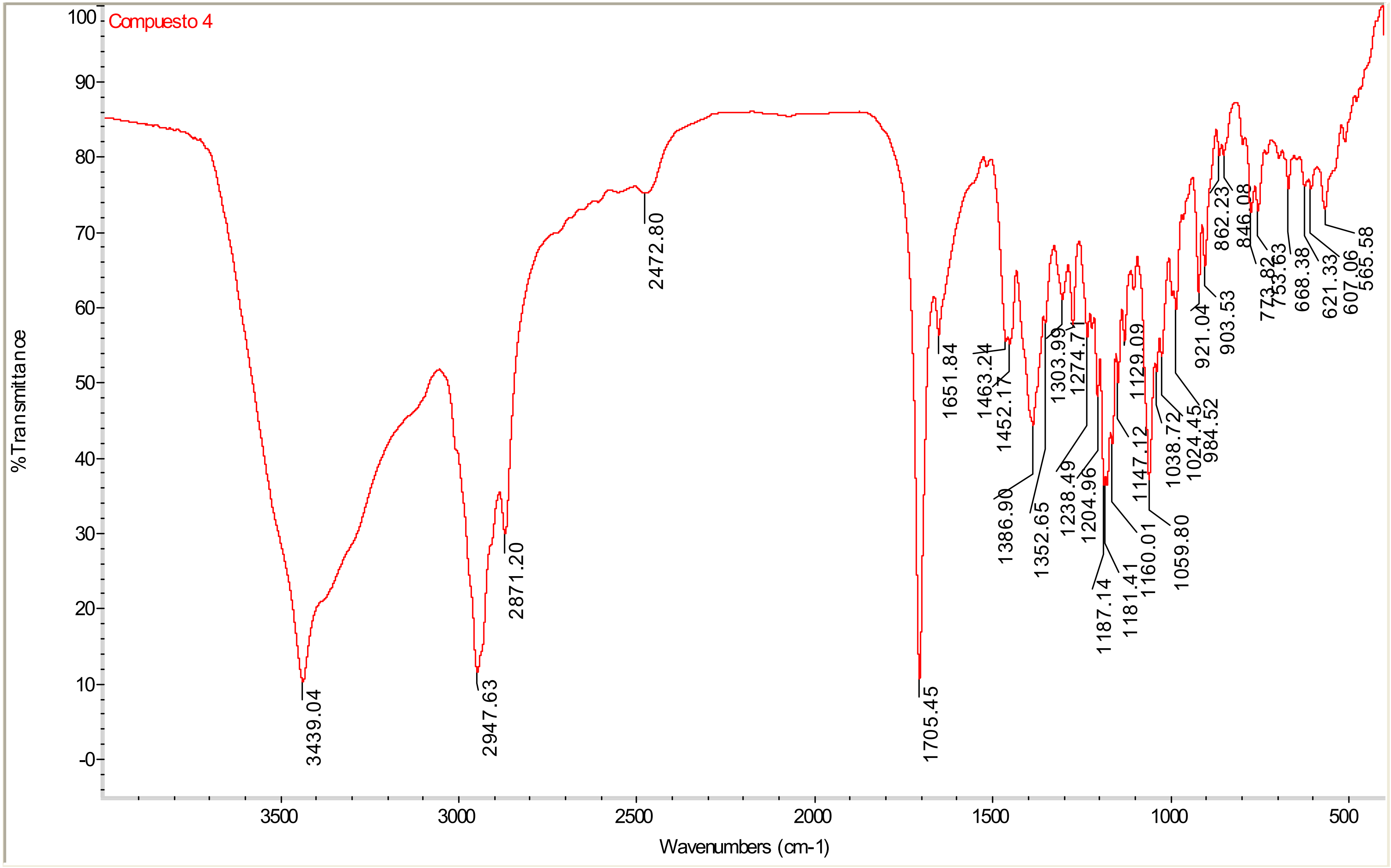

Figure S8. 1H- NMR spectrum of compound **5**.

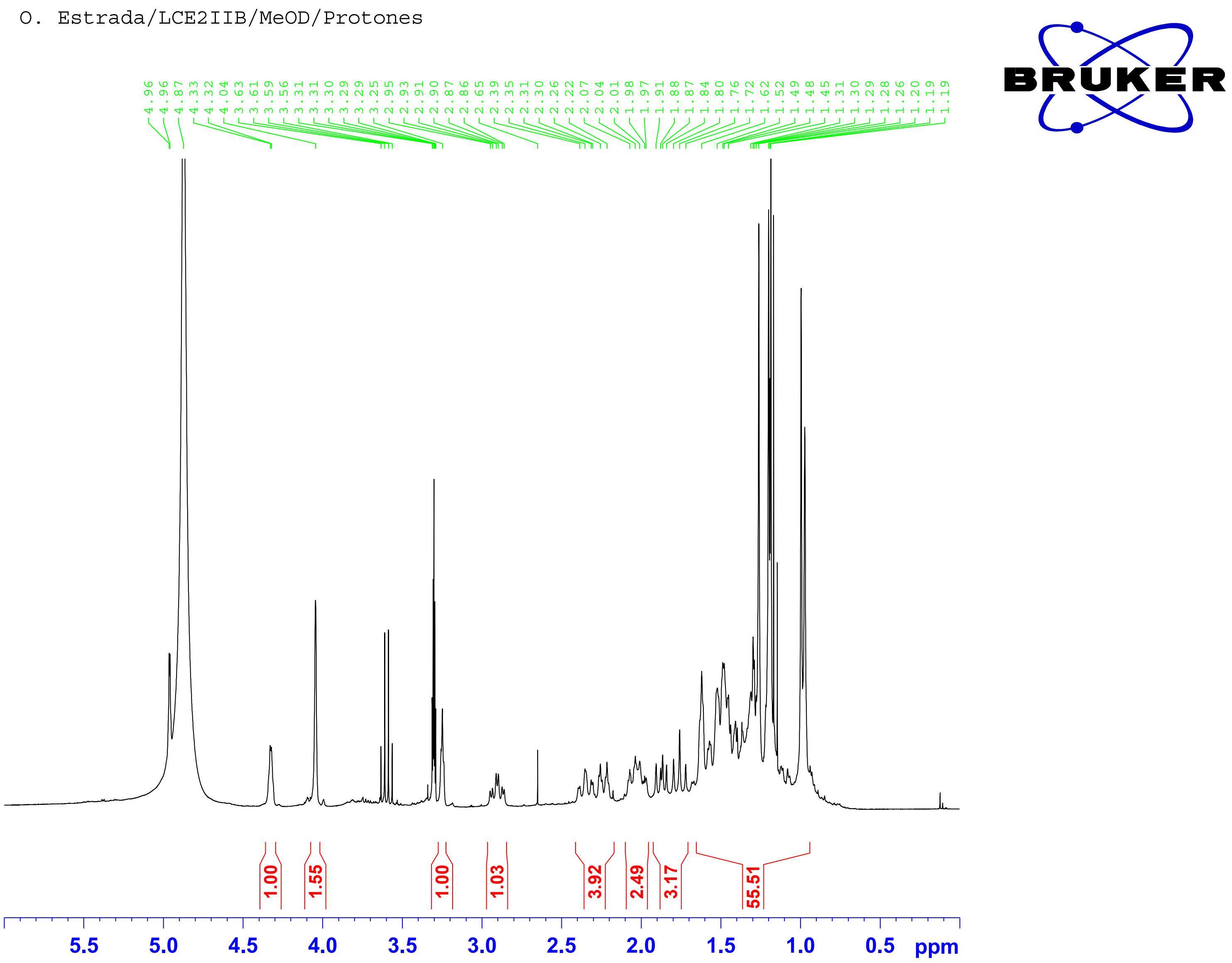

Figure S9. 13C-NMR spectrum of compound **5**.

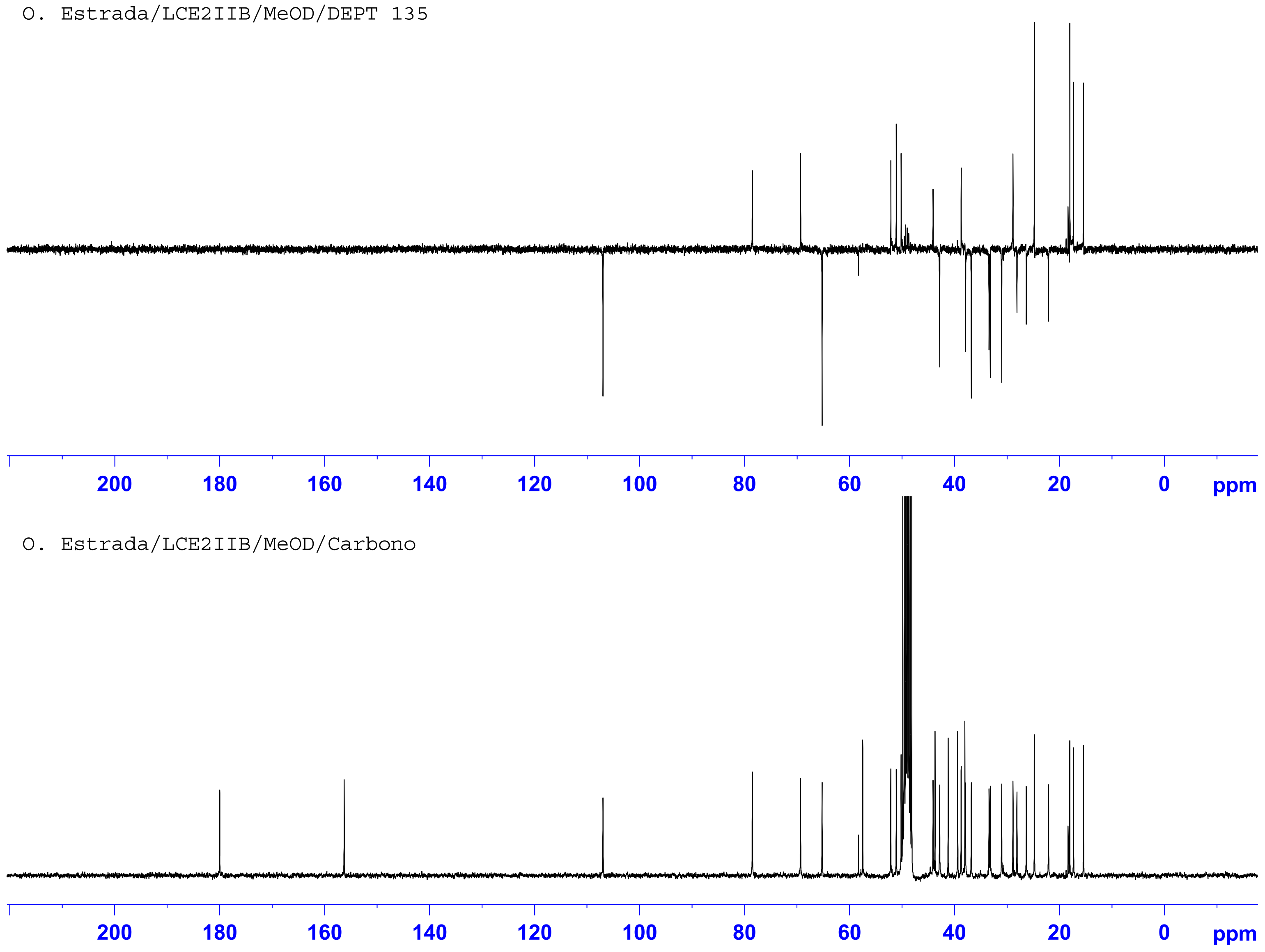

Figure S10. HMQC spectrum of compound **5**.

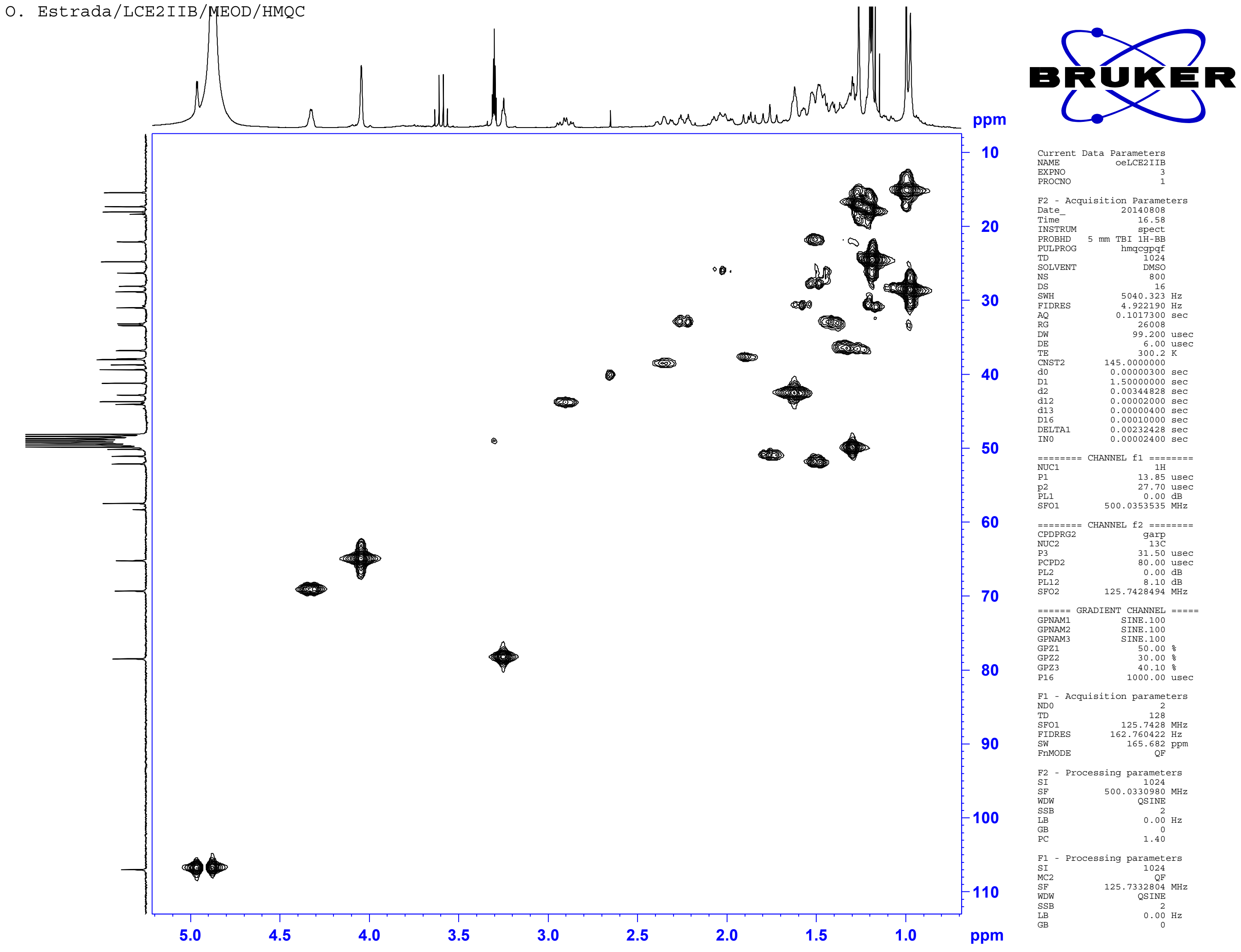

Figure S11. HMQC spectrum of compound **5**.

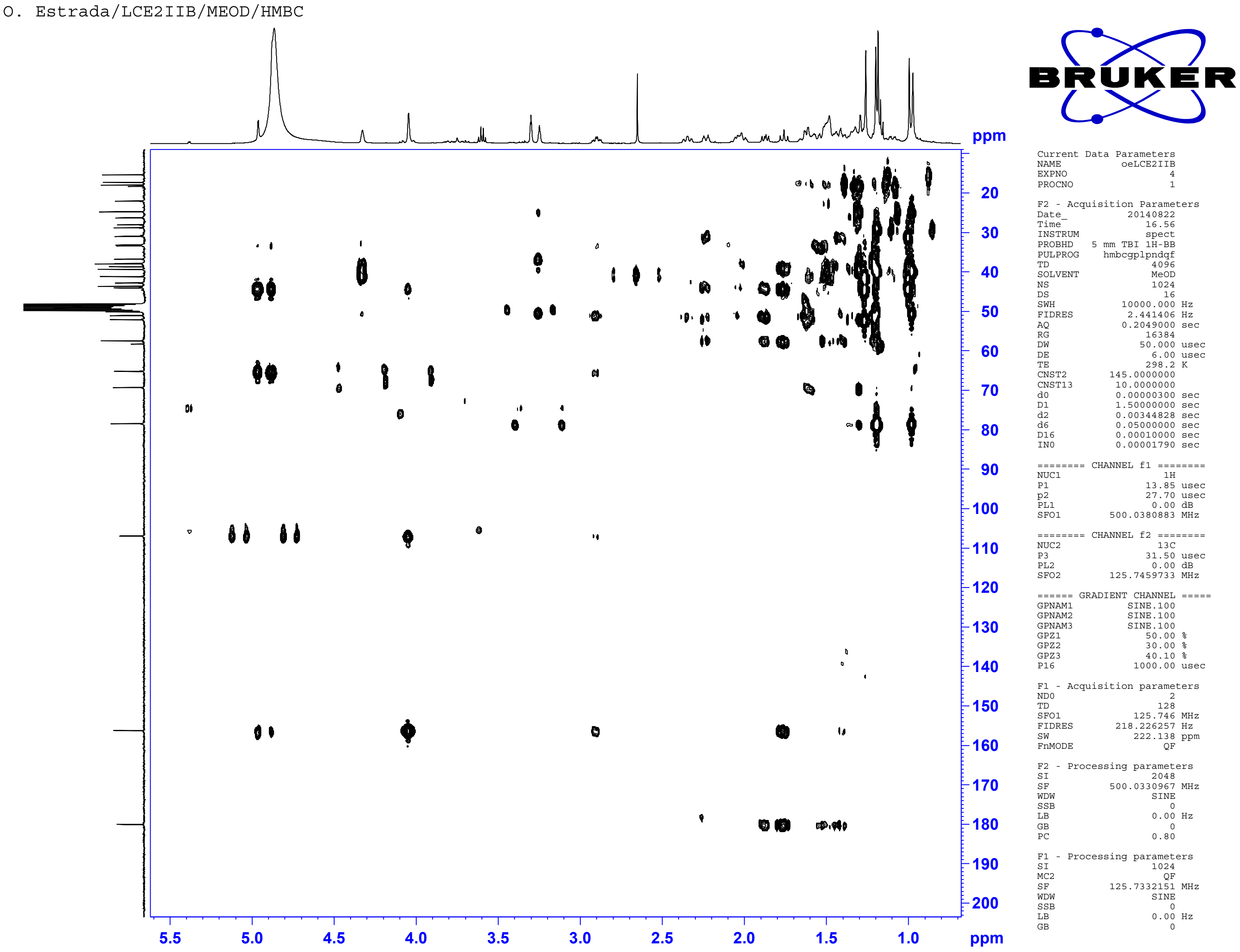

Figure S12. HPLC-MS of compound **5**.

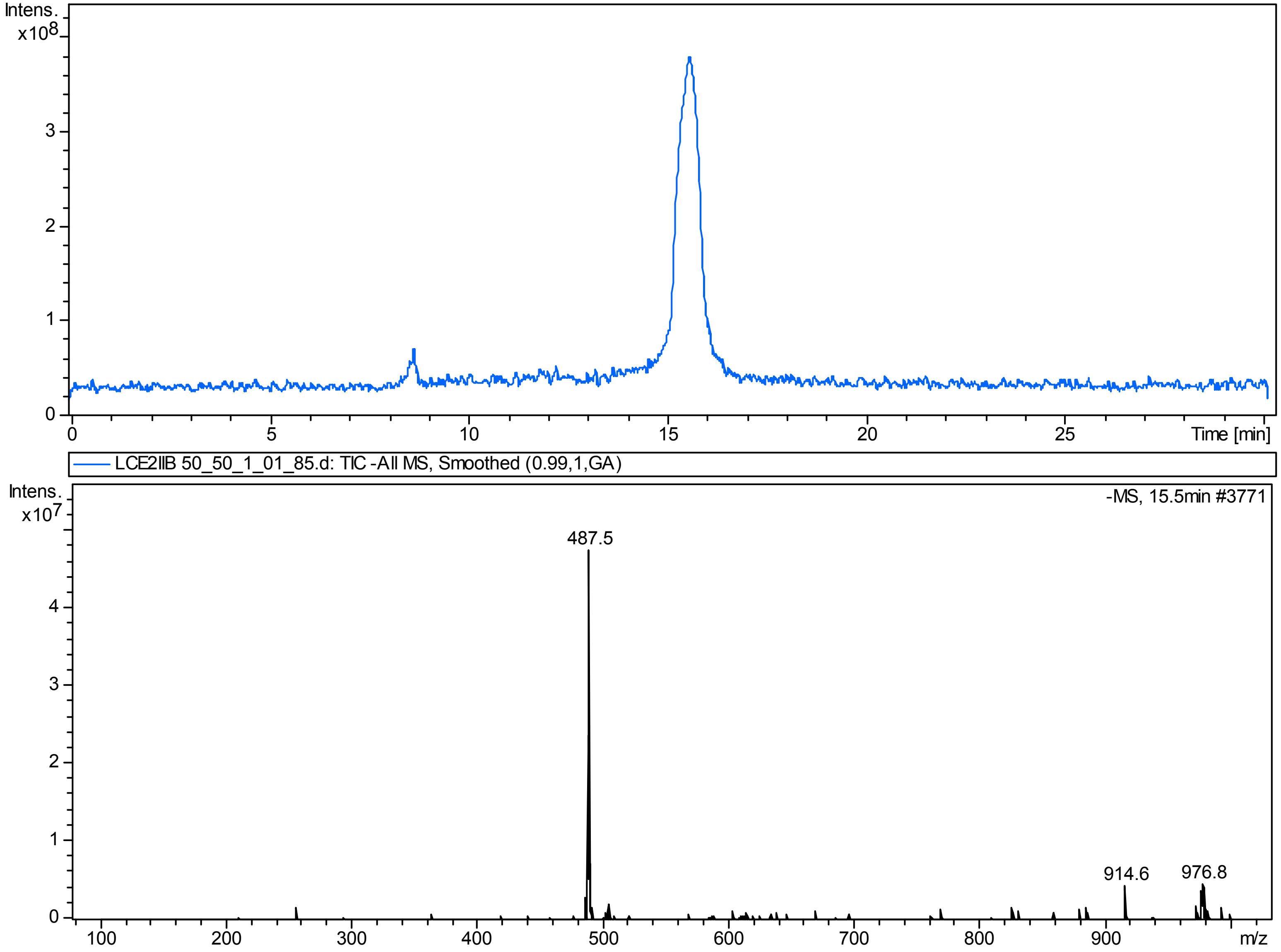

Figure S13. IR spectrum of compound **5**.

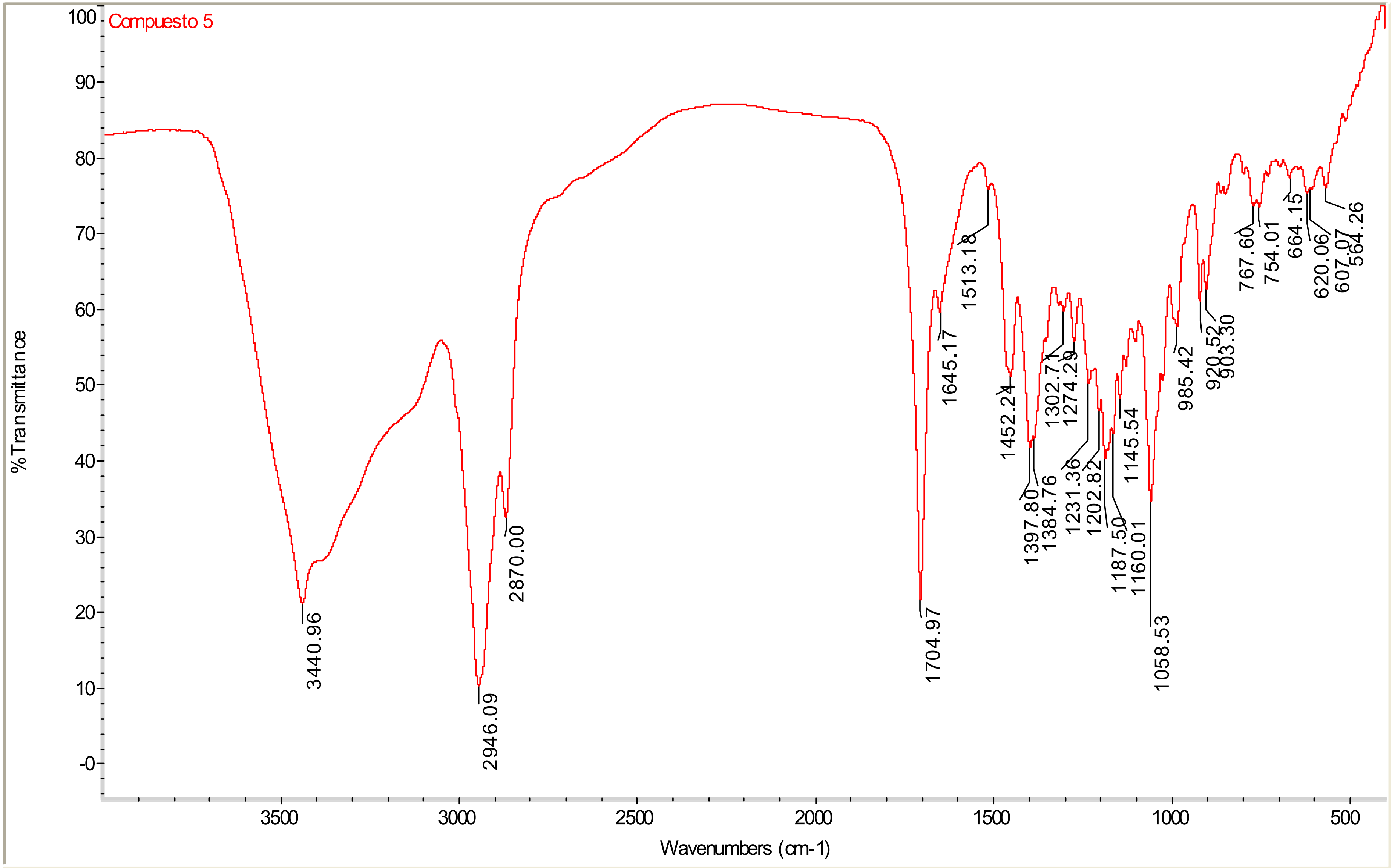

Figure S14. ^{1}H-NMR spectrum of compound **6**.

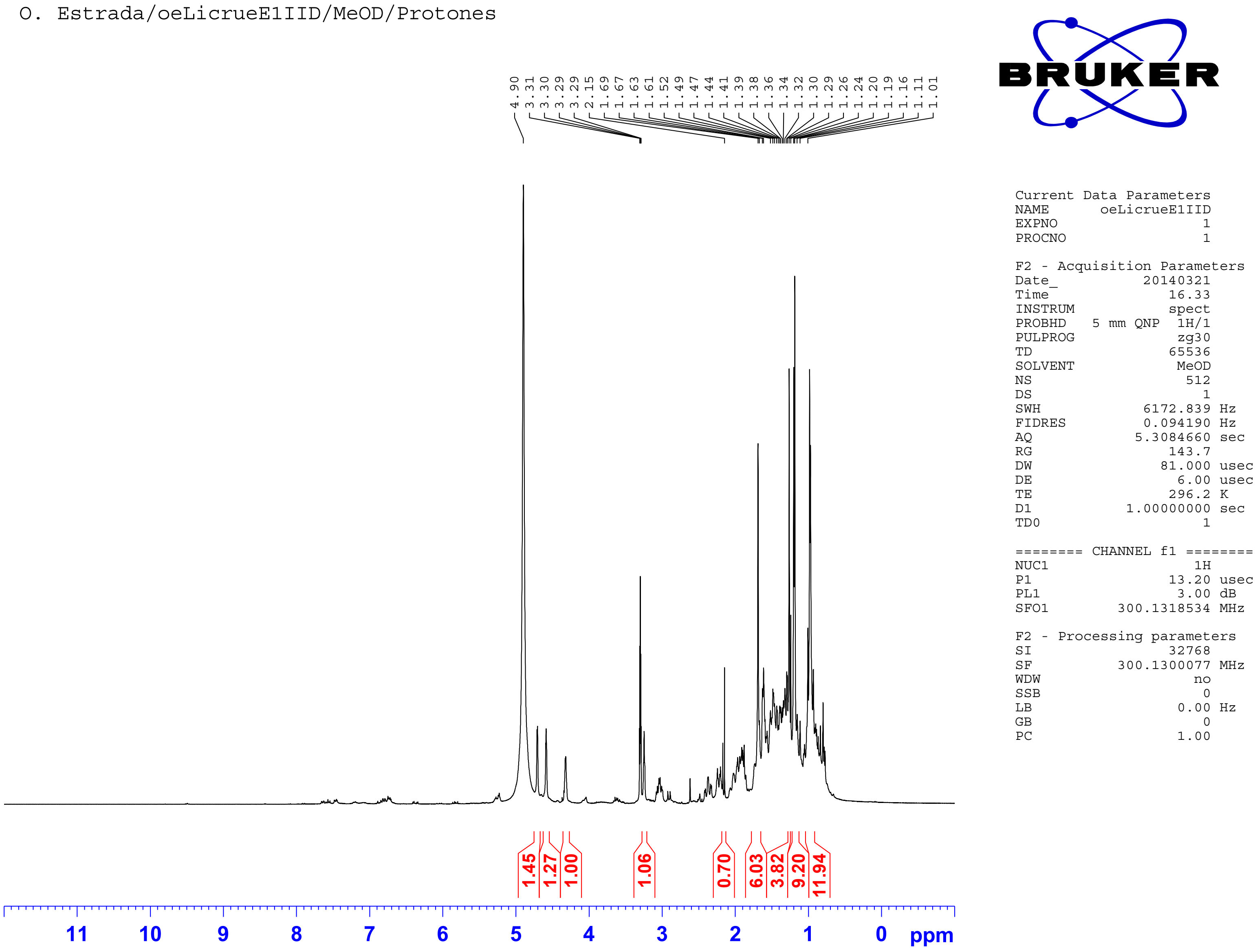

Figure S15. ^{13}C-NMR spectrum of compound **6**.

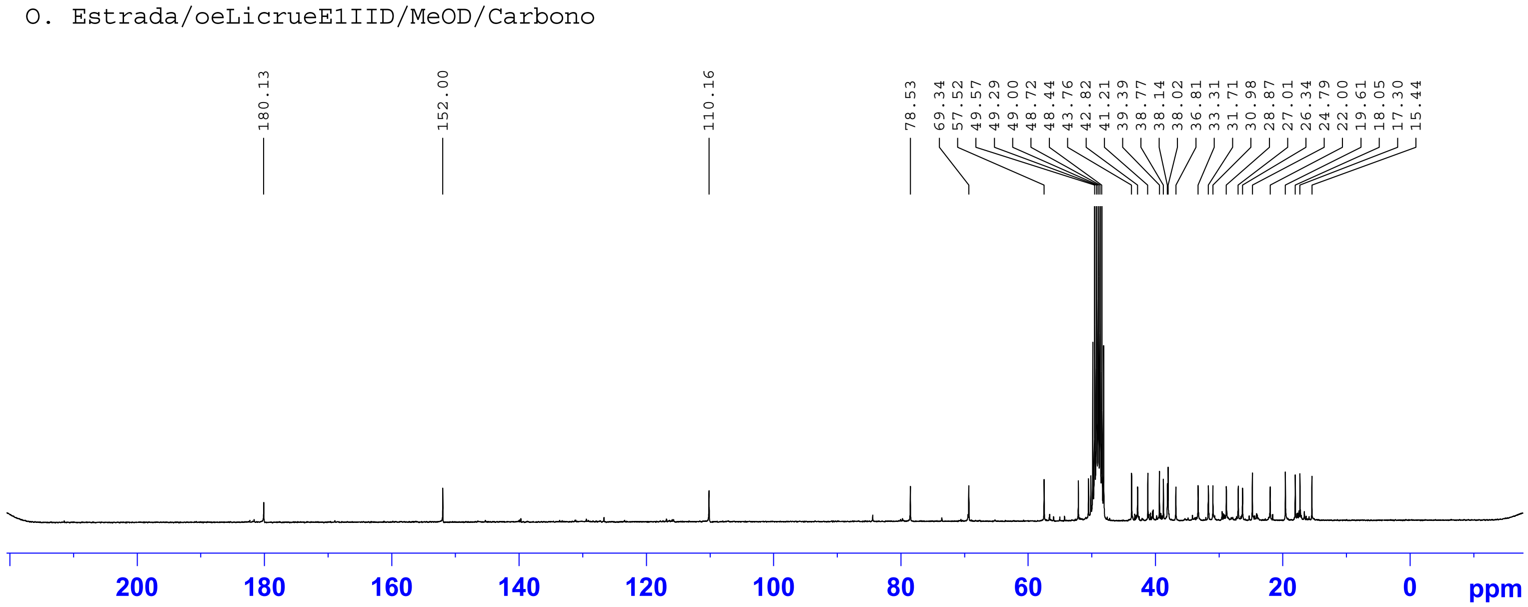

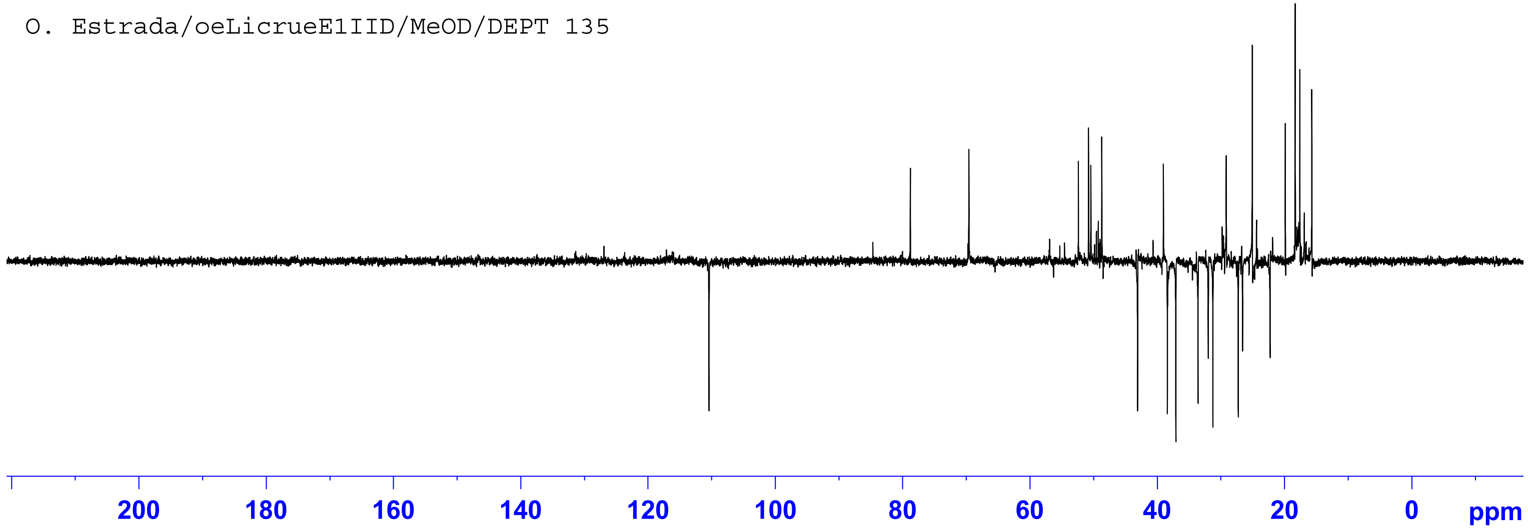

Figure S16. HPLC-MS of compound **6**.

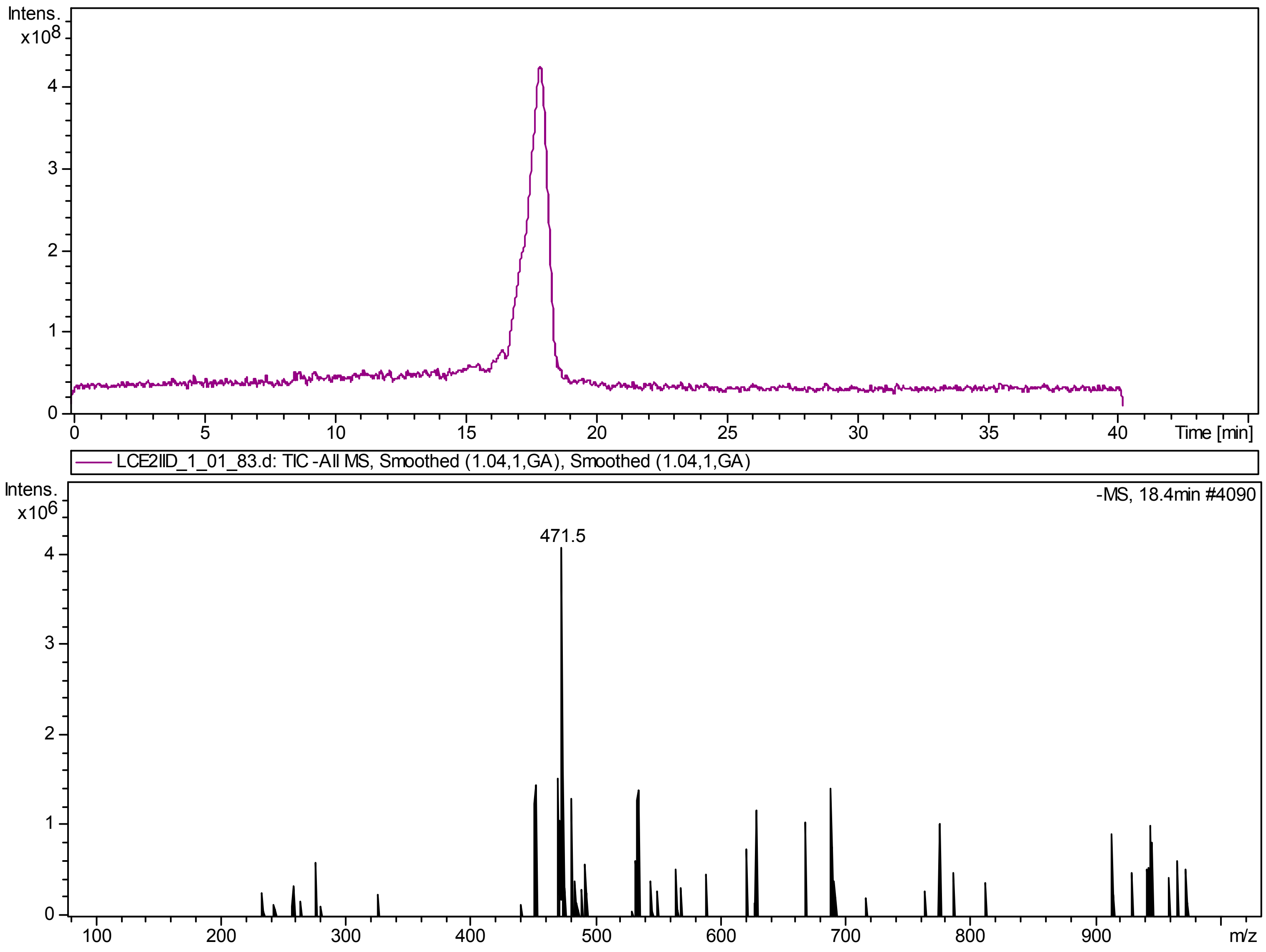

Figure S17. IR spectrum of compound **6**.

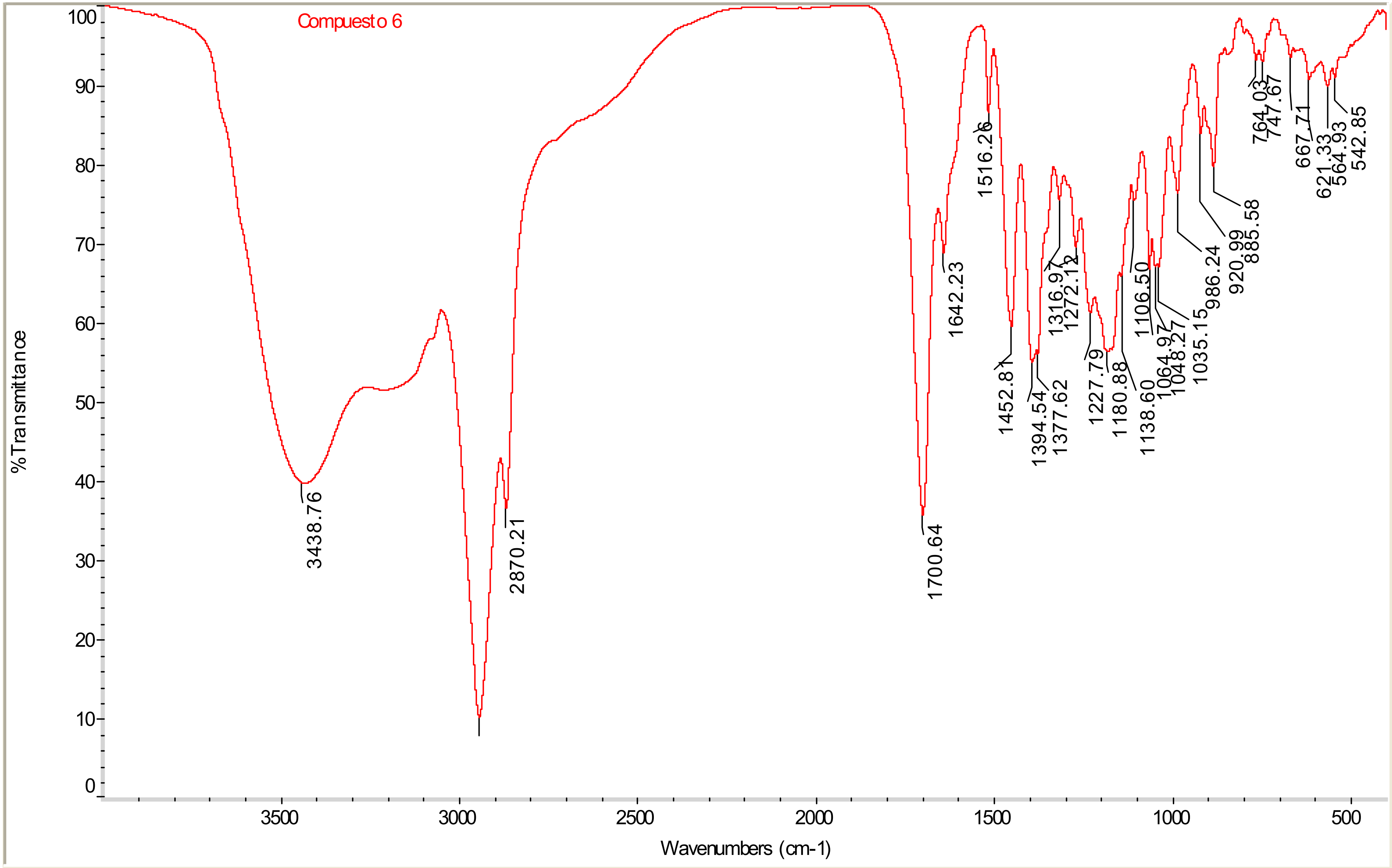

Supplement: Supplementary file 1 [file molecules-19-21215-s001.pdf]
